# Recent Trends and Developments in Graphene/Conducting Polymer Nanocomposites Chemiresistive Sensors

**DOI:** 10.3390/ma13153311

**Published:** 2020-07-24

**Authors:** Golnoush Zamiri, A. S. M. A. Haseeb

**Affiliations:** Centre of Advanced Materials, Mechanical Engineering, Faculty of Engineering, University of Malaya, Kuala Lumpur 50603, Malaysia

**Keywords:** graphene, conductive polymer, sensing material, resistive gas sensors

## Abstract

The use of graphene and its derivatives with excellent characteristics such as good electrical and mechanical properties and large specific surface area has gained the attention of researchers. Recently, novel nanocomposite materials based on graphene and conducting polymers including polyaniline (PANi), polypyrrole (PPy), poly (3,4 ethyldioxythiophene) (PEDOT), polythiophene (PTh), and their derivatives have been widely used as active materials in gas sensing due to their unique electrical conductivity, redox property, and good operation at room temperature. Mixing these two materials exhibited better sensing performance compared to pure graphene and conductive polymers. This may be attributed to the large specific surface area of the nanocomposites, and also the synergistic effect between graphene and conducting polymers. A variety of graphene and conducting polymer nanocomposite preparation methods such as in situ polymerization, electropolymerization, solution mixing, self-assembly approach, etc. have been reported and utilization of these nanocomposites as sensing materials has been proven effective in improving the performance of gas sensors. Review of the recent research efforts and developments in the fabrication and application of graphene and conducting polymer nanocomposites for gas sensing is the aim of this review paper.

## 1. Introduction

Graphene possesses unique properties including a high specific surface area (2630 m^2^g^−1^) and excellent electron mobility, and the atoms of a single-layer graphene sheet can adsorb gas molecules and provide the largest sensing area per unit volume, which makes it suitable as an active material for gas-sensing applications [1,2,3]. The interaction between graphene sheets can be variable from weak van der Waals interactions to strong covalent bonding [4]. These different interactions disturb the electronic balance of graphene, which can be readily monitored by convenient electronic techniques. Graphene indicates excellent high carrier mobility at room temperature due to the charge carriers of graphene having zero rest mass near its Dirac point [5].

Graphene is a p-type semiconductor that contains a larger hole to electron ratio concentration and has to pull an electron effect in a gas atmosphere [6]. When graphene absorbs the gas molecules, weak hybridization and coupling interactions generate between the graphene surface electron and the gas molecules, and they can only move up and down in small increments of the Fermi level. The Fermi level and consequently graphene conductivity will be changed by electron or hole doping. The relative position of the electron in orbit identifies the donor and the acceptor molecule. Gas molecules act as an electron donor when the Fermi in graphene is at a lower level than the valence band of adsorbed gas, and also when the Fermi in graphene is at a higher level, the gas molecules act as an acceptor [7,8,9]. All these are unique and attractive features of graphene, making it an ideal candidate for gas detection [10]. Therefore, great efforts have been put into the research and development of gas-sensing devices based on graphene and its derivatives. Nevertheless, sensors based on pure graphene sensors have some drawbacks because dangling bonds on their surface are too few to restrict the chemisorption of target molecules on the graphene surface [11]. Graphene tends to stack and self-aggregate because of the existence of strong π–π* interactions, van der Waals forces, and high surface energy, which leads to limited gas-sensing performance [12]. Incorporation of other nanomaterials including metal or metal oxide nanostructures and conducting polymers into graphene sheets prevents graphene from becoming agglomerated and, besides, cause a good distribution of nanostructures [13]. Because of low cost, simplicity, being compatible with modern electronic devices and their high sensitivity, metal oxide semiconductors have attracted a lot of attention. However, gas sensors based on metal oxide semiconductors generally have the disadvantage of poor selectivity between gases and also working at a high temperature which results in high consumption energy [14].

Conducting polymers, including polypyrrole (PPy), polyaniline (PANi), polythiophene (PTh), and so on, have been used as the sensing materials of gas sensors. Conducting polymer-based gas sensors are more sensitive, with a shorter response time at room temperature, that tune both chemical and physical properties by using different substituents in comparison with most commercially available metal oxide (MO)-based gas sensors. Another advantage of conducting polymers is their synthesizing procedure is facilitated through chemical or electrochemical processes. Also, copolymerization or structural derivations can modify their polymer molecular chain structure [15].

In recent years, the development of novel polymer nanocomposites has attracted significant worldwide research interest. The advantage of polymer-nanocomposite includes the value-added properties of the pure polymers without affecting their processability, inherent mechanical properties, and lightweight [16,17,18]. While both graphene and conducting polymers present some unique and exciting capabilities in the detection of a variety of gases, some researchers came up with the idea of mixing these materials to fabricate a graphene/conducting polymer composite with better sensing characterizations [19,20,21]. Several methods such as chemical, electrochemical, and physical techniques have been used to synthesize conducting polymers and graphene composites for fabricating chemiresistive gas sensors. The most widely used chemical technique for the synthesis of conducting polymers and graphene composites is in situ polymerization in a solution containing monomer and graphene [22].

Nowadays, many studies investigated the performance of gas sensor-based graphene and its composites with metal oxides and polymers [19,23,24,25,26,27]. Several review papers on graphene and graphene/conducting polymer nanocomposites-based gas sensors are available in the literature. A list of these review papers is given in Table 1.

A critical review of the published papers indicates that there is a gap in our knowledge about the comparison in preparation and sensing performance of graphene and different conducting polymers in the application of chemiresistive sensors for detecting various target gases. This article focuses on recent research efforts, developments, and approaches for the preparation of graphene and conducting polymer nanocomposites. The fabrication of chemiresistive gas sensors with graphene and conducting polymer nanocomposites is described along with a discussion of sensing performances.

## 2. Chemiresistive Gas Sensors

**Figure 1:**
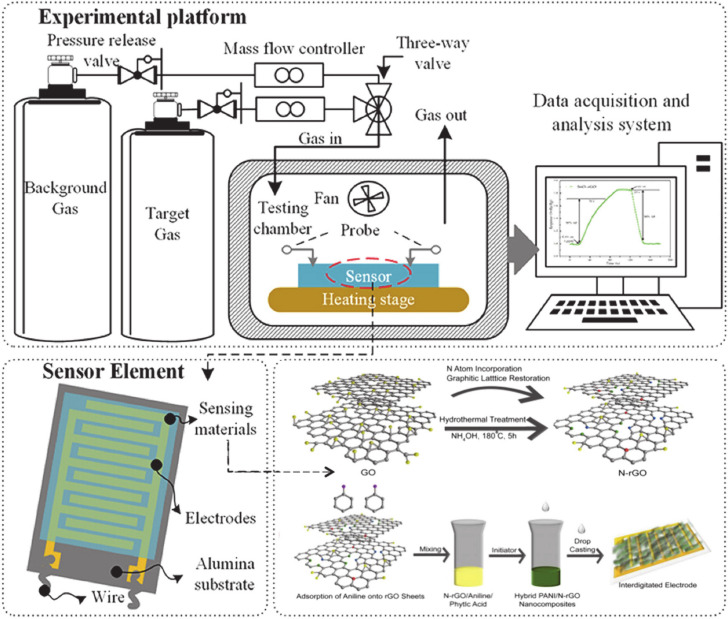
Schematic of electrodes and sensor devices (adapted from references [47,48]).

Sensors with accurate sensing performance have attracted much attention to monitoring and controlling emissions from various emitters [30]. Gas sensors consist of sensing material and are devices that can detect combustible, toxic gases, and oxygen depletion [3,31,32,33,34]. According to various kinds of reaction with external atmospheres, gas sensors can be classified into chemiresistors [35,36], silicon-based field-effect transistors (FET) [37], micro-electro-mechanical systems (MEMS) [38], surface work function (SWF) change transistors [39], surface acoustic wave (SAW) change transistors [2], and quartz crystal microbalance (QCM) sensors [2]. Among these options, resistive sensors are the most popular gas sensors due to their cheap fabrication process, smooth operation, and possible miniaturization [24]. A chemiresistive gas sensor measures the resistance changes of the sensing materials under target gas exposure. The schematic illustration of chemiresistive gas sensor is shown in Figure 1. Certain kinds of conducting materials experience a change in their electrical resistance in response to an interaction with gases and vapors [40]. Chemiresistive sensors can be utilized in several applications such as air-quality monitoring, medical diagnostics, detection of toxic and flammable gases, and food processing due to their excellent sensitivity, low cost, simplicity, and compatibility with modern electronic devices [41,42]. Since the discovery of chemiresistive-type sensors, metal oxide has always been used as sensing materials because of their benefits such as low cost and easy fabrication. Typical metal oxide semiconductors (MOS) that are widely used to detect harmful and toxic gases, include TiO_2_, Fe_2_O_3_, ZnO, SnO_2_, and WO_3_. MOS materials possess good sensing properties, but require high operating temperatures [24]. Theoretical and experimental results showed that graphene and its derivatives indicated a high specific surface area and good electron mobility [43]. On the other hand, conducting polymers have been used as the active layer of gas sensors since the early 1980s [44]. The sensors based on conductive polymers have many improved properties such as high sensitivities and short response time at room temperature, compare to the sensors made of metal oxides. According to different studies, the use of graphene and conducting polymer composites can improve the selectivity and other important sensing parameters of chemiresistive sensors which might be attributed to several factors such as synergistic and geometrical effects [23,43,45,46].

### 2.1. The Detection Mechanism of Chemiresistive Sensors

**Figure 2:**
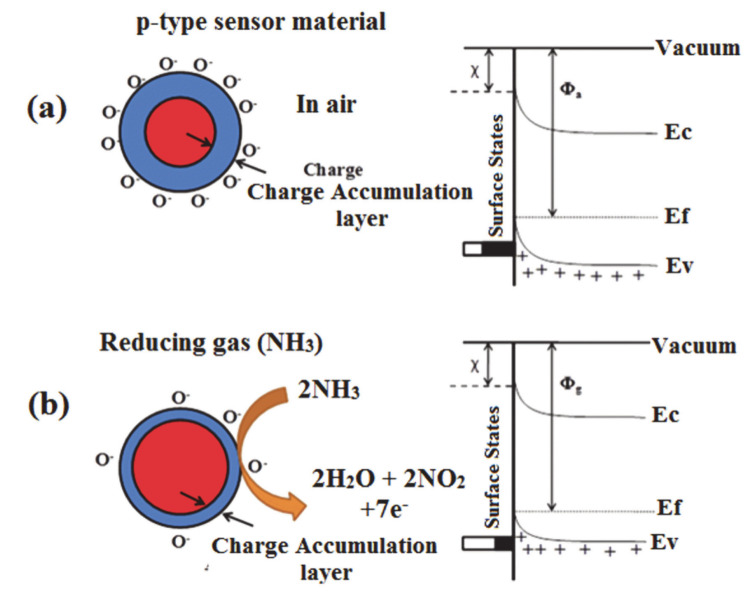
Interaction between (**a**) oxygen and sensing material; and (**b**) interaction between NH_3_ and sensing material (adapted from reference [51]).

To enhance the sensitivity and selectivity of a resistive gas sensor, it is essential to understand the sensing mechanism. It is well known that the conductance of n-type semiconductors increases with a reducing analyte and decreases with an oxidizing one [49]. The opposite effects are observed with a p-type semiconductor with holes being the majority charge carriers. The typical sensing mechanism of the p-type semiconducting sensor is shown in Figure 2. The conductivity increases in the presence of an oxidizing gas as the number of holes increases and decreases when a reducing gas is introduced as the hole charge carrier concentration decreases [50].

### 2.2. Gas-Sensing Performance Parameters

The sensing performance parameters of the gas sensor include response, sensitivity, selectivity, stability, repeatability, response and recovery time, the limit of detection, and working temperature [52]. The response of the sensor towards reducing gases is defined as the ratio of the resistance when exposed to the background and target gas environments. On the other hand, the response towards oxidizing gases is defined as the ratio of the resistance when exposed to the target gas and background environment. The response of the sensor is calculated by the following equations:

For reducing environments;

S = R_o_/R_g_ (1)

For oxidizing environments;

S = R_g_/R_o_ (2)

where, R_o_ and R_g_ are the resistances of the sensor in background gas and the presence of target gas, respectively, and S is the response of the sensor. Based on the electrical response, different approaches are used to determine the sensitivity of a gas sensor. Sensitivity is the change degree in response to a certain concentration of target gas. The ability of a sensor to selectively respond is known as selectivity. Selectivity refers to the characteristics that determine whether a sensor can respond selectively to an analyte or a group of analytes. Repeatability is how much the gas sensor test results will be constant when they are tested in the same environment continuously and whether it can affect the working life of the sensor. Response time and recovery time are other important parameters for determining the performance of a sensor towards a specific gas. The response time and recovery time are defined as the time to reach 90% of the total change in resistance during exposure and removal of the target gas, respectively. The lowest concentration of target gas that can be detected by the gas sensor is known as the limit of detection (LOD). The temperature that can give the gas sensors its highest sensitivity is known as the working temperature [23,53,54,55].

### 2.3. Sensing Material

To detect the target gases, variety of materials such as conducting polymers [56,57], carbon nanotubes [58] and MO, in varieties of forms (e.g., thick or thin films, nanorods, nanowires, etc. [59]) have been widely used. The most common gas sensors used in industries are MO semiconductor-based sensors owing to their high sensitivity and fast response time [60,61]. Although MO semiconductor gas sensors are highly sensitive, the poor selectivity, short lifetime, and high operating temperature are the drawbacks of these sensors [62,63]. These drawbacks limit the application of MO as a sensing layer in the gas sensors and bring other alternatives such as graphene and conducting polymers to the center of the researchers’ attention [11,19,64].

Nowadays, graphene and its derivatives have received attention owing to their unique properties such as good conductivity, large specific surface area, feasible adsorption of gas molecules, and their potential to be modified by functional groups as a sensing material for gas sensor fabrication [43,65]. On the other hand, conducting polymers, including PPy, PANi, PTh, etc. which have high sensitivities, short response time, and suitable mechanical properties have been used as the active layers of gas sensors since the 1980s [66,67,68,69].

Several studies reported that graphene and conducting polymer composites indicated excellent mechanical, thermal, gas barrier, electrical, and flame retardant properties in comparison with pure conducting polymers [70,71,72,73,74].

## 3. Graphene/Conducting Polymer Nanocomposites for Chemiresistive Gas Sensor Application

Over the last few years research on incorporating graphene into polymer matrices to provide novel nanocomposite materials with enhanced electrical, thermal, mechanical, electrical, and other properties due to the large aspect and surface-to-volume ratios of the nanofiller has reseived extensive attention [75,76,77,78,79]. Different techniques have been reported for the preparation of graphene-polymer nanocomposites including in situ polymerizations [80,81,82,83], electro-polymerization [84], solution mixing [85,86], self-assembly approaches [87,88], and so on [89].

The in situ polymerization involves chemical reactions. Normally in this technique, the nanofiller mixes with monomers in a solvent. The use of monomers can help to adjust the interactions between materials which makes possible intercalation and results in exfoliation and also compared to high molecular weight polymers, monomers diffuse into the galleries of the silicate more efficiently [90,91]. Although in situ polymerization method has advantages in promoting effective dispersion of nanofillers in polymer matrices, this technique has some disadvantages such as complex procedures and processing steps and also requires expensive reactants [92]. On the other hand, it is only applicable for the limited elastomers and thermally unstable polymers which are insoluble in the solvent. Another widely used method to prepare graphene and conducting polymer composites is solution mixing due to it being amenable to small sample sizes and possessing a low-viscosity condition for dispersing the nanofiller. This technique is considered an effective means to prepare composites with uniformly dispersed graphene or its derivatives. Despite the advantages of the solution mixing method, the removal of the solvent which normally remains on the graphene after several washing and drying processes is a big issue [93]. Electropolymerization is a novel and convenient method to fabricate graphene and polymer composites. This kind of method has many advantages, such as being a short process, easier to control, and eco-friendly [22]. The electrochemical polymerization method consists of a three-electrode system including, the counter, reference, and working electrodes. During the polymerization process, an anodic potential is applied to the monomer to oxidize onto the electrode. However, as fairly large electrode potential is a necessity for the oxidation of aniline, the consumption of other substances is restricted [94]. The self-assembly approach is one the most important techniques to fabricate materials in nano, micro and macro scales and is an efficient way to control the composition of a composite. In this method, molecules are utilized for building complex molecular architecture under eco-friendly conditions. However, the self-assembly approach also exhibits some drawbacks, such as the difficulty in achieving high quantities of materials, the corresponding high costs, and in some cases, purification limitations [95,96,97]. The advantages and disadvantages of each method are summarized in Table 2.

**Figure 3:**
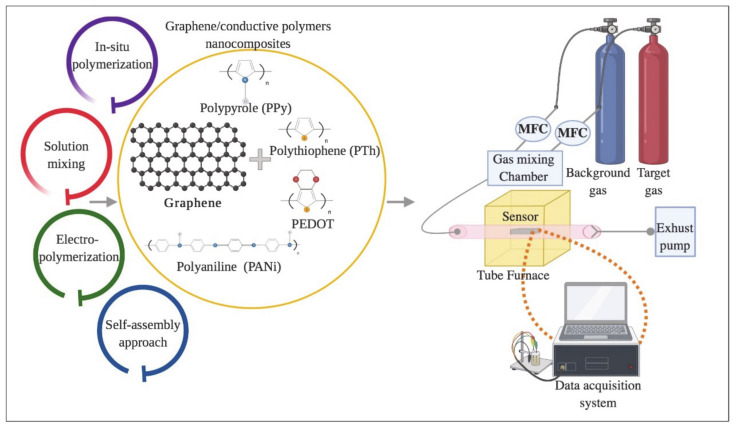
Schematic for preparing graphene/conductive polymers composites and their application for gas sensing.

This section focuses on the synthesis of graphene and different conductive polymers (such as PPy, PANi, PTh, poly (3,4 ethyldioxythiophene) (PEDOT), etc.) nanocomposites and their application for gas sensing (Figure 3).

### 3.1. Graphene/Polyaniline (PANi) Nanocomposites

#### 3.1.1. Preparation of Graphene/Polyaniline Nanocomposites

The most widely used methods to synthesize graphene and PANi nanocomposites for the application of gas sensors are in situ chemical polymerization and solution mixing. In situ chemical polymerization was reported as an efficient method to uniformly disperse graphene oxide (GO) with a strong interaction between the GO and polymer matrix and [98]. Typically, the reduced graphene oxide (rGO) aqueous solution was added in 1 mL of aniline in 50 mL of aqueous 1 M HCl solution quickly. The anilinium cation grows on the surface of rGO and after that, the aniline monomers were polymerized by the addition of ammonium peroxydisulfate (APS) [75,99,100,101,102,103,104,105].

**Figure 4:**
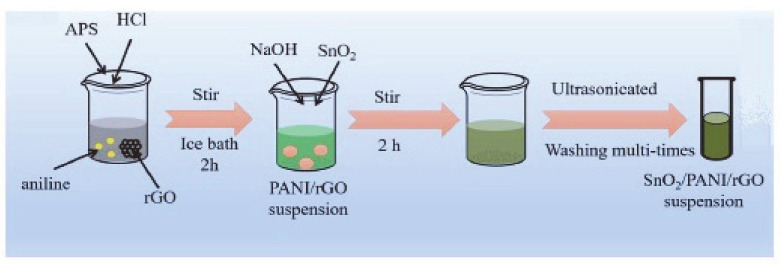
The preparation process of in situ polymerized SnO_2_/polyaniline (PANi)/rGO nanocomposite (adapted from reference [109]).

In situ chemical oxidative polymerization also was used for the preparation of graphene quantum dots (GQDs)/PANi [106,107]. SnO_2_/rGO/PANi nanocomposite was synthesized by using the in situ polymerization technique [108,109,110]. As seen in Figure 4, rGO and aniline were added into the mixture of HCL and distilled water. PANi/rGO composite was formed when the color of the solution changed from white to green. SnO_2_ powder and NaOH were added in the same solution and then stirred to obtain the precipitate of SnO_2_/rGO/PANi composite [109].

**Figure 5:**
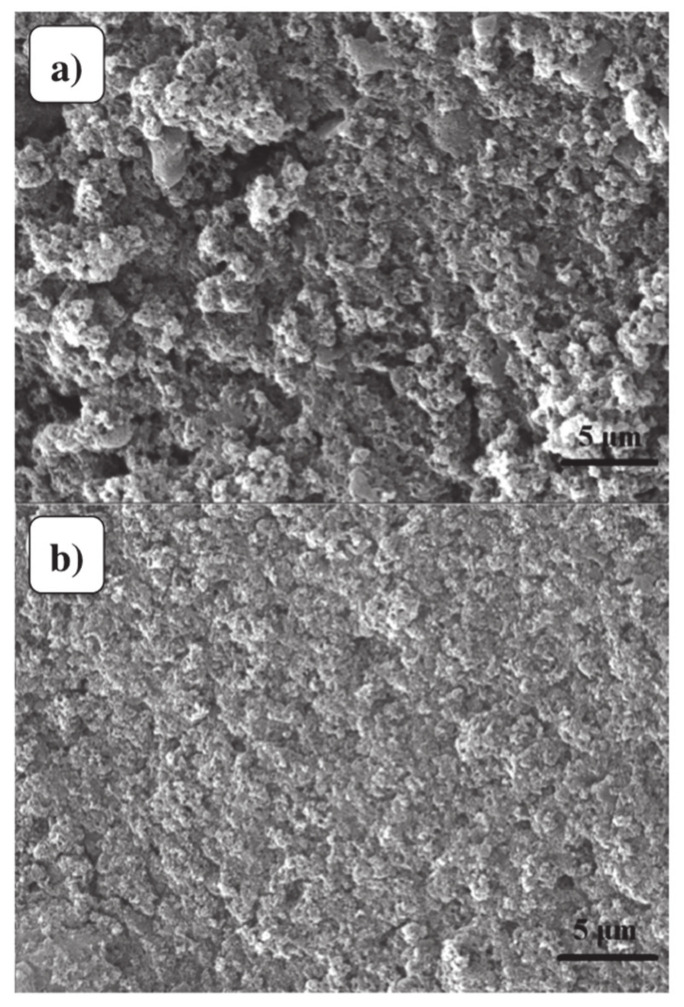
(**a**) Scanning electron microscopy (SEM) images of (**a**) pure PANi, and (**b**) S, N: GQDs/PANi hybrid sensing films (adapted from reference [106]).

The S, N: GQDs were prepared by the hydrothermal method in the presence of citric acid and thiourea. Ammonium persulfate (APS) was added to the mixture of aniline, 30 mL hydrochloric acid (HCl) and then transferred to the suspension of citric acid and thiourea. In the last step, the powder of S, N:GQDs/PANi hybrid was washed and dried in the oven. Figure 5a,b present scanning electron microscopy (SEM) images of pure PANi and S, N: GQDs/PANi hybrid, respectively, and the images illustrate the uniform nanofibrous structure. The SEM image of prepared S, N:GQDs/PANi hybrid (Figure 5b), shows that the nanofibrous structure of PANi remained the same, and all the S, N: GQDs were homogeneously surrounded by PANi [106].

Junfeng Tian, et al. synthesized TiO_2_/GO/PANi by using the in-situ polymerization of aniline in the presence of TiO_2_/GO nanocomposite. Firstly, TiO_2_/GO composite was mixed with 50 mL of deionized water under stirring, and then aniline was added to the solution drop by drop in an ice water bath to obtain a hybrid material consisting of bulk reduced TiO_2_/GO/PANi [111].

The most simple process method to prepare graphene and PANi nanocomposite is solution mixing [89,112]. Normally in this technique, as-prepared graphene and PANi are mixed under stirring [113]. To prepare graphene/PANi composite by solution mixing, PANi was treated with ammonia (NH_4_OH) solution firstly and then dissolved in N-methyl-2-pyrrolidone (NMP) while stirring. After that, graphene was added to the solution to make graphene-PANi nanocomposite [85,114].

#### 3.1.2. The Sensing Performance of Gas Sensors Based on Graphene/PANi Nanocomposites

The NH_3_ sensor based on rGO–PANi hybrid, which was synthesized using a simple, chemical oxidative polymerization method and coated on a flexible polyethylene terephthalate (PET) thin film was reported by Shouli Bai [101]. The combination of the functionalized rGO with PANi developed a new sensing material with high sensing characteristics compared to the constituent counterparts. The sensor based on rGO–PANi hybrid indicated the highest response of 344.2 under 100 ppm NH_3_ exposure, good selectivity to some of the volatile organic compounds (VOCs) tested, and short response time and recovery time which were 20 s and 27 s, respectively at room temperature. GO-rambutan like PANi hollow nanosphere hybrid (GPA) for the detection of ammonia gas was prepared by the in situ chemical oxidation polymerization method and assembled on PET substrates as flexible devices [115]. The sensor-based on GPA indicated a response value of around 31.8 toward 100 ppm NH_3_, response and recovery time (102 s and 186 s, respectively) and low detection limit of 50 ppb.

Jaber Nasrollah Gavgani, et al. [106] and M. Hakimi, et al. [107] reported NH_3_ sensors based on (S, N-doped GQDs)/PANi loading on PTE and N-doped GQDs/PANi hybrid assembled on two different electrodes, silver (Ag) and aluminum (Al). The composite of S, N: GQDs and PANi lead to significant improvement in the response (~42% and 385% under 100 ppm and 1000 ppm NH_3_ exposure, respectively), and response and recovery time (115 s and 44 s, respectively) at room temperature [106]. The NH_3_ gas sensor based on N-GQDs/PANi with Ag contact illustrated the best response of 110.92 compared to the sensor with an Al electrode (86.91) under 1500 ppm target gas at room temperature [107].

The rGO@ SnO_2_/PANi composites were prepared by using the in situ chemical oxide polymerization for detecting different gases such as NH_3_ [108], CH_4_ [110], and H_2_S [109] at room temperature. The rGO@ SnO_2_/PANi composite film exhibited 160% response to 20 ppm NH_3_, 9.1% toward 100 ppb H_2_S, and 26.1% to 100 ppm of CH_4_.

The Gr/PANi nanocomposites were prepared by solution mixing [114] and in situ polymerization [104] for detecting toluene (C_6_H_5_–CH_3_) gas indicated the response of 11.6% and 90% toward 100 ppm and 5000 ppm C_6_H_5_–CH_3_ gas, respectively.

**Figure 6:**
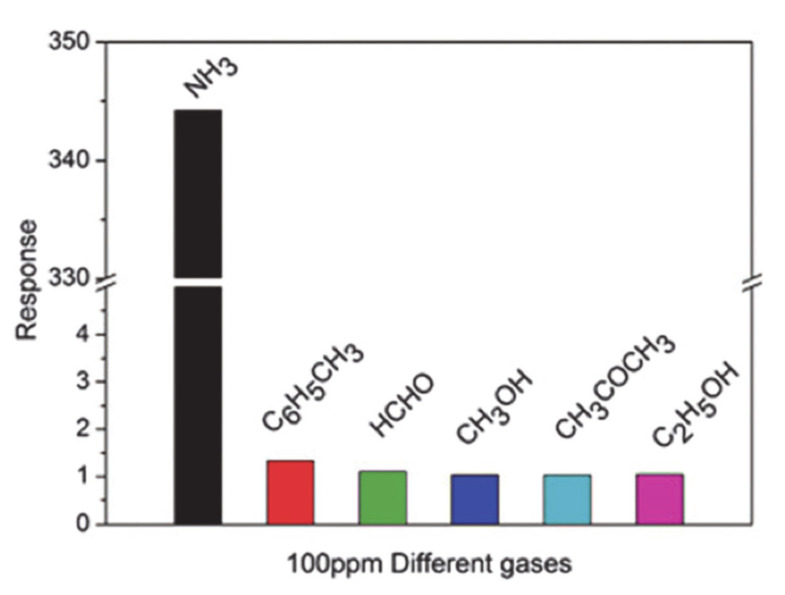
The selectivity of rGO–PANI hybrid thin films to 100 ppm of different gases. (adapted from reference [101]).

Table 3 critically investigates and lists the pieces of literature that have been studied the graphene/PANi composites and their sensing performance under various target gases such as NH_3_, CH_4_, C_6_H_5_–CH_3_, Benzene, and H_2_S. Among different NH_3_ gas sensors based on graphene and PANi nanocomposites, rGO–PANI hybrids loaded on a flexible PET thin film indicated the highest response of 344.2 to 100 ppm NH_3_ and the response time and recovery time were 20 s and 27 s, respectively [101]. The selectivity data of the rGO-PANi hybrid under 100 ppm ethylbenzene, methanol, formaldehyde, ethanol, and acetone exposure at room temperature are indicated in Figure 6 and it shows the high-selectivity response to 100 ppm NH_3_. The enhancement of the sensing performance of the sensor based on rGO-PANi hybrid under NH_3_ exposure might be related to the acid-base deprotonation process of PANi nanoparticles, resulting in the selective response to NH_3_ gas.

The enhancement of NH_3_ gas sensor based on rGO/PANi hybrid sensing performance can be caused by the acid-base de-doping process of PANi nanoparticles and the synergetic effects between rGO and PANi [57]. This novel composite can be used as a sensing layer for the detection of different gases, but as it can be understood from this table, it was mostly used for the detection of NH_3_.

### 3.2. Graphene/Poly (3,4 Ethyldioxythiophene) (PEDOT) Nanocomposite

#### 3.2.1. Preparation of Graphene/PEDOT Nanocomposite

**Figure 7:**
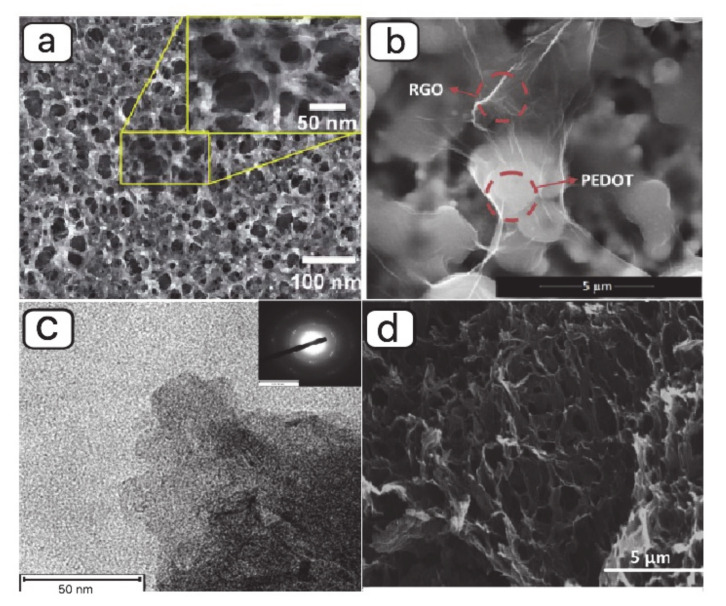
SEM images of (**a**) porous poly (3,4-ethylene dioxythiophene) (PEDOT) layer deposited on rGO Langmuir–Blodgett (LB) films (adapted from reference [118]), (**b**) PEDOT/rGO film (adapted from reference [120]), (**c**) transmission electron microscope (TEM) image of graphene–PEDOT:poly (styrenesulfonate) (PSS) nanocomposite (adapted from reference [121]), and (**d**) SEM image of CuTSPc@3D-(N)GF (adapted from reference [122]).

Different synthesis techniques have been reported for synthesizing graphene and poly (3,4-ethylene dioxythiophene) (PEDOT) nanocomposite. Yajie Yang, et al. [90,118] prepared rGO and porous conducting polymer (PEDOT) nanostructure by in situ polymerization technique. Figure 7a shows the SEM image of the porous PEDOT layer coated on rGO Langmuir–Blodgett (LB) films. The rGO layer is prepared from the GO LB deposition and a thermal reduction treatment [119]. The in situ polymerization of 3,4-ethylene dioxythiophene (EDOT) monomer was used to deposit PEDOT nanostructure on the surface of rGO sheets. Figure 7b indicates the SEM image of PEDOT/GO films prepared by a fully electrochemical route and reported by Katarzyna Dunst and his group [120]. The electro-polymerization and electrochemical reduction of PEDOT/GO were accomplished in GO aqueous solution and 0.1 M KCl at a constant potential, respectively. Yotsarayuth Seekaew, et al. [121] synthesized graphene–PEDOT: poly (styrenesulfonate) (PSS) nanocomposite by using solution mixing method (Figure 7c). Hamed Sharifi Dehsari, et al. [79] investigated sensing performance of NH_3_ gas sensor based on copper (II) tetrasulfophthalocyanine supported on a 3-dimensional nitrogen-doped graphene-based framework (CuTSPc@3D-(N)GF)/(PEDOT-PSS) nanocomposite (Figure 7d).

PEDOT:PSS is a widely used conjugated polymer due to its excellent electrical conductivity, high transparency, good processability, and low redox potential [123,124]. Firstly, PEDOT:PSS was dissolved in the mixture of dimethyl sulfoxide (DMSO), ethylene glycol (EG) and triton x-100 to prepare the graphene–PEDOT:PSS ink, and then stirred at room temperature. To synthesize graphene solution, as-prepared graphene powder was mixed with 5 mL of DMSO. Then, graphene solution was added to 40 mL of PEDOT:PSS. To prepare GQDs/PEDOT-PSS compound, Mahdieh Hakimi, et al. [125] combined the PEDOT-PSS and N-GQDs solutions in which N-GQDs was synthesized using a hydrothermal technique in the presence of citric acid and urea.

#### 3.2.2. The Sensing Performance of Gas Sensors Based on Graphene/PEDOT Nanocomposites

PEDOT, one of the most famous conducting polymers, attracted researchers’ attention due to its good conductivity, electrical properties associated with its low bandgap, and good stability [126,127]. The conducting polymer nanostructures such as PEDOT plays an important role in enhancing the sensing properties of graphene and its derivatives [128,129]. Yajie Yang, et al. synthesized a single layer of RGO and PEDOT nanocomposite using LB deposition and an in situ polymerization technique and as they reported, the RGO and PEDOT composite indicated better sensing performance to NO_2_ gas compared to the sensor based on pure rGO [90]. The electro-polymerization technique was used to fabricate a sensing PEDOT/RGO layer and reported by Dunst and his group in Gdansk University of Technology using [120] and the effect of annealing temperature on the sensing performance of the film under NO_2_ were also investigated. The PEDOT/RGO composite showed good sensing performance to NO_2_, higher operating temperature, and improved sensitivity. The gas sensor based on (CuTSPc@3D-(N)GF)/(PEDOT-PSS) nanocomposite indicated better response (5 and 53 times) and lower response and recovery times towards 200 ppm of NH_3_ compared with pure PEDOT-PSS and CuTSPc@3D-(N)GF [122]. 

As we can see in Table 4, PEDOT/RGO nanocomposites are mostly utilized as a sensing material for detecting NH_3_ and NO_2_ gases. The gas-sensing performance of PEDOT/rGO nanocomposites prepared by in-situ polymerization technique revealed in contrast to other PEDOT/rGO nanocomposites, which were listed in Table 4 exhibited excellent sensing performance as well as response and recovery time to NO_2_ gas [90].

**Figure 8:**
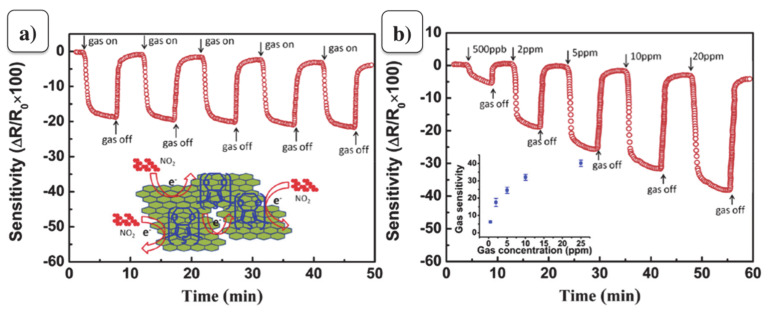
(**a**) Repeatability of sensor based on rGO and PEDOT nanocomposite and (**b**) sensitivity of sensor based on rGO and PEDOT nanocomposite under various NO_2_ concentrations (adapted from reference [90]).

The repeatability of the gas sensor based on rGO and PEDOT composite is illustrated in Figure 8a. Five response cycles of the sensor based on rGO and PEDOT nanocomposite under 2 ppm NO_2_ gas exposure have been executed repeatedly. Yang et al. [90] concluded that the sensor exhibits excellent repeatable properties, and the response levels of the sensor can be maintained after repeated cycles. Moreover, the composite of rGO and PEDOT provides excellent reproducing stability toward lower concentration of NO_2_ gas, because of the synergistic effect between rGO and PEDOT. The gas sensor based on rGO and PEDOT nanocomposite under the various concentrations of NO_2_ gas (Figure 8b), shows a fast response and recovery even at the ppb level.

### 3.3. Graphene/Polypyrrole (PPy) Nanocomposites

#### 3.3.1. Preparation of Graphene/PPy Nanocomposites

**Figure 9:**
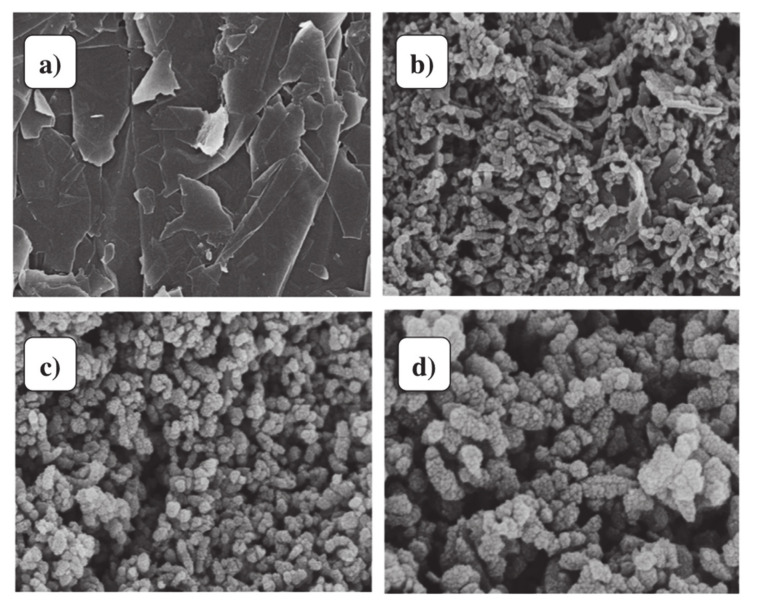
SEM images of (**a**) GNs, (**b**) PPy–GN, and (**c,d**) TiO_2_@PPy–GN (adopted from reference [77]).

Highly stable rGO/PPy nanocomposites were synthesized using in situ polymerization of pyrrole monomers onto rGO [130]. Firstly, GO was reduced by hydrazine and then pyrrole polymerized at high temperatures using an oxidizing agent and a surfactant. Wang-De Lin, et al. [131] prepared graphene/PPy by the chemical oxidative polymerization method. Normally in this technique, pyrrole was mixed with graphene solution contained distilled water and 1% cetyltrimethylammonium bromide (CTAB), then sonicated for 10 min. On the other hand, distilled water contained 1% APS was added to the solution during the sonication. Cuili Xiang, et al. [77] decorated graphene/PPy nanocomposite with titanium dioxide (TiO_2_) nanoparticles utilized the sol-gel method to obtain TiO_2_@PPy–graphene nanosheet (GN) nanocomposite (Figure 7b). Figure 9a–d display the SEM images of GNs, PPy–GN, and TiO@PPy–GN, respectively. As seen in Figure 9a, the morphology of graphene nanosheets is normally like irregular plates with smooth surfaces. Figure 9b illustrates PPy nanofibers coated the GNs. After decorating PPy and graphene composite by TiO_2_ nanoparticles, it can be observed that the surface of the TiO_2_@PPy–GN nanocomposite became rough and TiO_2_ nanoparticles homogeneously dispersed into the nanocomposite (Figure 9c–d).

#### 3.3.2. The Sensing Performance of Gas Sensors Based on Graphene/PPy Nanocomposites

PPy is one of the most widely used conducting polymers in different applications due to its high conductivity, facile synthesis process, and great environmental stability [132]. Graphene and PPy nanocomposites had also become appealing sensor materials due to their combined effects, and better electrochemical performance compared with pure PPy and graphene [133]. Rawoof A. Naikoo and Radha Tomar [134] investigated a CO gas sensor using Zeolite-X/reduced graphene oxide/polypyrrole (Na-X/rGO/PPy) nanocomposite as a sensing material. When they increased the concentration of CO gas, the response of gas sensor based on Na-X/rGO/PPy composite was increased from 14.9% to 77.4%. Huynh Ngoc Tien and Seung Hyun Hur [135] fabricated NO_2_ gas sensors based on RGO–PPy composite which exhibited high sensitivity (~32%) when exposed to 50 ppm of NO_2_ at room temperature. The gas sensor based on RGO-PPy composite decorated by TiO_2_ indicated a response of 102.2% toward 50ppm of NH_3_ [77].

The investigation of gas sensors based on RGO-PPy composites toward various target gases such as NO_2_, humidity, NH_3_, and CO is indicated in Table 5. The comparison of chemiresistive gas sensors’ performance based on graphene/PPy nanocomposites under different gases displays that the gas-sensing performance of rGO/PPy nanocomposite exhibited the highest response of 102.2% under 50 ppm NH_3_ exposure when it is decorated with TiO_2_ nanoparticles [77]. TiO_2_ is an n-type semiconductor material with a bandgap of around 3.33 eV, which made it useful for sensing materials [136]. Development of a gas sensor that can operate at room temperature and at the same time retain the sensing properties of TiO_2_ nanoparticles can be a useful way to enhance sensing performance of rGO/PPy.

### 3.4. Preparation and Sensing Performance of Gas Sensors Based on Other Graphene-Based Polymer Composites

Polystyrene (PS)-modified graphene composites were prepared by using the solution blending method [70,137,138]. The composite thin films exhibited semi-conducting behavior in nature. PS/graphene nanosheet (GNS) composites were prepared by Hu et al. [139] utilizing an in situ polymerization technique. The thermal stability and electrical conductivity of the nanocomposite were higher compared to the pure PS.

**Figure 10:**
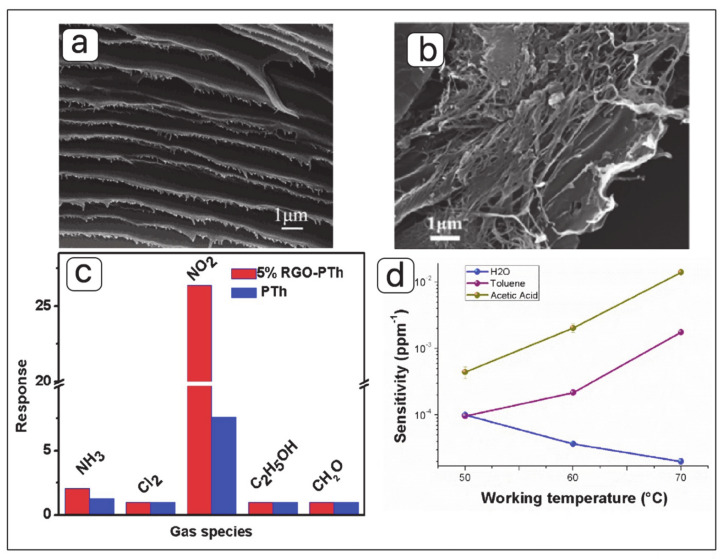
SEM images of (**a**) polydiacetylene (PDA)/graphene film (adapted from reference [140]), and (**b**) rGO-polythiophene (PTh) hybrid, (**c**) response of sensors based on PTh and rGO-PTh to different gases at room temperature (adapted from reference [141]), and (**d**) sensitivity values changes of PS and polyhydroxibutyrate (PHB) doped with 5,10,15,20-tetraphenylporphyrin (H_2_TPP) and mesoporous graphene to water vapors, toluene and acetic acid (adapted from reference [142]).

Figure 10a displays an SEM image of polydiacetylene (PDA)/graphene composite which was prepared by the self-assembly approach [140]. PDA monomers were mixed with chloroform and filtered and then this was dropped on to graphene and exposed to air to evaporate the solvent. Diacetylenic moieties were polymerized under ultraviolet (UV) light with a wavelength of 254 nm at room temperature and after polymerization the white sample became blue. The VOC sensor based on PDA/graphene composite indicated high sensitivity and short response time in the concentration range of 0.01 to 10%.

The in situ polymerization technique was used to prepare a hybrid of ethylenediamine-modified, rGO, and PTh and then the as-prepared hybrid was deposited on a flexible PET film to fabricate a sensor for NO_2_ detection [141]. PTh attracted researchers’ attention due to their characteristics of the inherently porous structure, remarkable environmental stability, and easy preparation [143,144]. The field-emission scanning electron microscope (FESEM) image of rGO-PTh hybrid (Figure 10b) confirms the successful combination between PTh and rGO. As shown in Figure 10c, hybridization of PTh with graphene enhanced response of gas sensor (26.36 under 10 ppm of NO_2_ exposure) compared to the pure PTh and graphene which might be attributed to synergistic effects between PTh and graphene [90,144,145,146,147].

Joshua Avossa, et al. [142] designed a nanofibrous conductive chemical sensor based on two insulating polymers (PS and PHB, named as PsB) doped with H_2_TPP and graphene which were selected for being versatile, biodegradable, eco-compatible, recyclable (PS) [148,149] and resistant to thermal excursions. The sensor indicated non-linear relationships between the conductivity and the temperature (Figure 10d). This means that, when the temperature increased, the electrical conductivity increased.

Rey Alfred G. Rañola, et al. [150] investigated graphene/polystyrene-sulfonate (rGO/PSS) nanocomposite prepared by solution mixing as a chemiresistive gas sensor for detecting trimethylamine (TMA). The sensitivity of the gas sensor was 1.72 × 10^−3^ ΔR/R mg L^−1^ and the LOD of the sensor was 22.67 mg/L.

**Figure 11:**
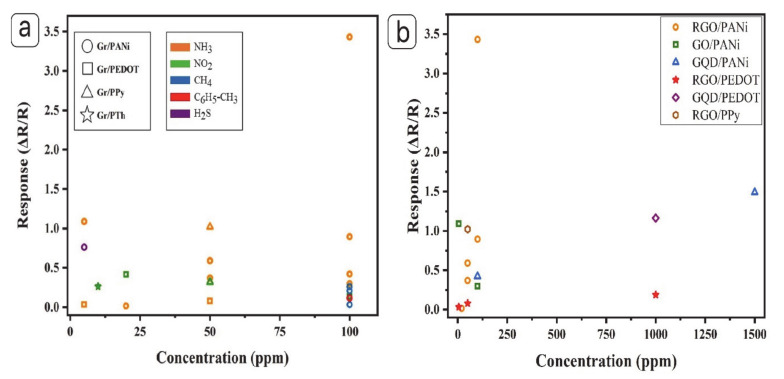
(**a**) Response versus different target gases concentration and (**b**) response versus ammonia concentration for gas sensors based on graphene and conductive polymer nanocomposites (schematic visualization of Table 3, Table 4 and Table 5).

Figure 11a indicates compiled results on sensing performance of gas sensors based on different graphene and conducting polymer composites under various target gases. In the past few years, the hybridization of conducting polymers by graphene and its derivatives has become important because of tunable morphology, high electrical conductivity, and the synergistic effect between graphene and conducting polymers might contribute to enhancing the gas-sensing performance of hybrids [11]. Among different target gases, graphene and its composite with conducting polymers show better sensing performance toward NH_3_ exposure. Figure 11b exhibits the response of various nanocomposites gas sensors for detecting different concentrations of NH_3_ (1–1500 ppm). As we can understand from the tables and Figure 9a,b, the composites of PPy, PANI, PTh, and PEDOT with graphene and its derivatives have been investigated during these years. However, PANi and PPy have a higher number of studies which might be attributed to the relatively better sensing performance [151]. Based on the collected results, graphene/PANi nanocomposite was investigated by Shouli Bai shows the highest response compared to other conducting polymers [101]. PANi, among different conducting polymers, has attracted a lot of attention due to its unique conduction mechanism, environmental stability, and facile synthesis and processability. Nanocomposites based on graphene and PANi presented special characteristics, such as excellent electrical conductivity, good thermoelectric properties, significant electrocatalytic activity, and great electrochemical stability. The enhancement of sensing properties for the graphene and PANi nanocomposite can be attributed to the synergistic effects between the graphene and the PANi. Besides, another factor to enhance gas-sensing performance is doping. Compared to other conducting polymers, composite based on PANi normally illustrates better gas-sensing performance which might be attributed to its reversible doping mechanisms. Oxidation or protonation process can help to improve the electronic structure and electrical properties of PANi which allow PANi to specifically detect oxidizing or reducing gas. Furthermore, this can also be the reason why PANi has achieved importance in comparison with other conducting polymers [152].

## 4. Conclusions

The preparation methods and sensing performance of graphene and conductive polymer nanocomposites are discussed in this review paper. The most widely used methods to prepare nanocomposites of graphene and conductive polymers for the application of gas sensors are in situ chemical polymerization. Mixing these two materials exhibited better sensing performance compared to pure graphene and conductive polymers because of the large specific surface area of the nanocomposites, which might be attributed to the anchoring of conducting polymers on the surface of graphene sheets. According to the tables listed, rGO–PANI hybrids loaded on a flexible PET thin film indicated the highest response of 344.2 to 100 ppm NH_3_, the gas-sensing performance of PEDOT/RGO nanocomposites prepared by in-situ polymerization technique revealed in contrast to other PEDOT/RGO nanocomposites exhibited excellent sensing performance to NO_2_, and rGO/PPy nanocomposite indicated the highest response of 102.2% under 50 ppm NH_3_ exposure when it is decorated with TiO_2_ nanoparticles. Compared to various gas sensors based on graphene and conductive polymer nanocomposites such as graphene/PEDOT, graphene/PPy, graphene/PTh, and so on, graphene and PANi nanocomposite gas sensor shows the highest response for detecting NH_3_ gas. The enhancement of gas accessibility might contribute to the large surface area of the composite materials and also the combination of materials with various properties can result in a synergistic effect. Incorporating the merits of high conductive graphene sheets with the advantages of conducting polymers has attracted researchers’ attention to tap their novel characteristics because of the synergistic effect between them. The superior electrical properties of graphene materials, including high carrier mobility, and detectable change in their resistance after adsorption or desorption of gases, also contribute to the high sensitivity.

The most important mechanism that should be considered in graphene and conducting polymer composites is normally referring to non-interface dependent complementary behavior, which is known as a synergistic effect. A synergistic effect determines when various constituents in a composite material are separately in contact with the gas phase and serve a different purpose that is complementary of the other constituents in a material.

The chemiresistors fabricated using the composite of graphene and other conducting polymers to date have received relatively little attention. On the other hand, there are only a few works that have studied the sensing performance of graphene/conductive polymer composites under various gases. Very few works have been reported on new preparation methods of this novel material and their application in gas sensors. The lower limit of detection for the given gas was not reported in most studies.

Improving the large-scale production of gas sensors based on graphene/conductive polymer nanocomposites, enhancing the selectivity by functionalizing the sensing layer, and increasing the stability of the active layer should be considered in future studies.

## Figures and Tables

**Table 1:** List of review papers on graphene and graphene/conducting polymer nanocomposites-based gas sensors.

Year of Publication	Title of Paper	Main Emphasis	References
2019	Review—Conducting Polymers as Chemiresistive Gas Sensing Materials: A Review	Conducting polymers	[28]
2018	A review on chemiresistive room temperature gas sensors based on metal oxide nanostructures, graphene and 2D transition metal dichalcogenides.	2D transition metal dichalcogenides, metal oxide nanomaterials, and graphene	[24]
2018	Research progress of gas sensor based on graphene and its derivatives: a review.	Graphene and its derivatives	[25]
2018	Graphene and its sensor-based applications: A review.	Graphene and its application in 3 different sensor applications such as electrochemical, strain and electrical sensors	[26]
2017	Chemo-electrical gas sensors based on conducting polymer hybrids.	Conducting polymers and conducting polymer hybrids for chemo-electrical gas sensors	[21]
2016	A review on graphene-based gas/vapor sensors with unique properties and potential applications.	Graphene	[23]
2015	Graphene–metal oxide nanohybrids for toxic gas sensor: A review.	Graphene and metal oxide hybrids	[27]
2015	Graphene-based hybrids for chemiresistive gas sensors	Graphene and graphene-based hybrids	[11]
2015	Elaborate chemical sensors based on graphene/conducting polymer hybrids.	Graphene and conducting polymer hybrids for chemical sensors	[29]
2014	Conducting polymer composites with graphene for use in chemical sensors and biosensors.	Chemical sensors and biosensors	[19]

**Table 2:** Summary of different fabrication techniques.

Fabrication Methods	Advantages	Disadvantages
In situ polymerization	Highly effective and a high level dispersion	Needs highly cost reactants and the processing steps are complicated
Solution mixing	Versatile and a good dispersion	Solvent removal is a critical issue
Electro-polymerization	Short process, easier to control, and eco-friendly	Available monomers are less
Self-assembly approach	Efficient way to precisely control the composition of a composite	High costs, purification limitations, and difficult to achieve a high quantity of materials

**Table 3:** Comparison of chemiresistive gas sensors performance based on graphene/PANi nanocomposites.

Target Gas	Sensing Material	Response	LOD	Response Time	Recovery Time	Nanocomposite Preparation Method	Ref
NH_3_	CRG/PANi	37.1% (50 ppm)	-	18 min	-	In-situ polymerization	[99]
	RGO–PANi	59.2% (50 ppm)	-	18 min	4 min		[116]
	(S, N: GQDs)/PANi	42.3% (100 ppm)	1–1000 (ppm)	115 s	44 s		[106]
	GS–PANi	1.6 (20 ppm)	71 ppb	11 min	-		[108]
	GO-PANi	R_g_/R_a_ = 30.8 (100 ppm)	50 ppb	102 s	186 s		[115]
	TiO_2_/GO/PANi	R_g_/R_a_ = 110 (100 ppm)	5 ppm	32 s	17 s		[111]
	N-GQDs/PANi	R_g_/R_a_ = 150.09 (1500 ppm)	-	366 s	4.98 s		[107]
	Py-RGO/PANi	59.1% (50 ppm)	0.2 ppm	-	-		[117]
	rGO–PANi	R_g_/R_a_ = 344.2 (100 ppm)	-	20 s	27 s		[101]
	NiNPs@3D-(N)GFs	750.2% (1000 ppm)	45 ppb	95 s	32 s		[83]
CH_4_	PANi/GO	20.9 (100 ppm)	-	3–120 s	3–120 s		[103]
	SnO_2_@rGO/PANi	26.1% (100 ppm)	-	-	-		[110]
	G/PANi-C15	3.25% (100 ppm)	10–1600 (ppm)	85 s	45 s		[100]
C_6_H_5_–CH_3_	C-PANi	11.6% (100 ppm)	-	8 min	22 min	Solution mixing	[114]
	PANi–G	90% (5000 ppm)	-	8.6 s	16 s	In-situ polymerization	[104]
Benzene	PANi–G	80% (5000 ppm)	-	16.25 s	18.5 s		
H_2_S	SnO_2_/rGO/PANi	76.25% (5 ppm)	50 ppb	80 s	88 s		[109]

**Table 4:** Comparison of chemiresistive gas sensors performance based on graphene/PEDOT nanocomposites.

Target Gas	Sensing Material	Response	LOD	Response Time	Recovery Time	Nanocomposite Preparation Method	Ref
NH_3_	PEDOT/RGO	3.43% (5 ppm)	200 ppb	90–100 s	180 s	In situ polymerization	[118]
	G/PEDOT:PSS	18.9% (1000 ppm)	10 ppm	3 min	5 min	Solution mixing	[121]
	GQDs/PEDOT-PSS	116.38% (1000 ppm)	-	7.7 min	10 min		[125]
	CuTSPc@3D-(N)GF/PEDOT-PSS	8% (50 ppm)	1–1000 ppm	138 s	63 s		[122]
NO_2_	PEDOT/RGO	41.7% (20 ppm)	-	170–180 s	70 s	In situ polymerization	[90]
		14% (100 ppm)	-	8.3 min	16.3 min	Electro-polymerization	[120]

**Table 5:** Comparison of chemiresistive gas sensors performance based on graphene/PPy nanocomposites.

Target Gas	Sensing Material	Response	LOD	Response Time	Recovery Time	Nanocomposite Preparation Method	Ref.
NO_2_	RGO/Polypyrrole (PPy)	32% (50 ppm)	-	-	-	In situ polymerization	[135]
Humidity	RGO/PPy	138%	-	15 s	20 s		[131]
NH_3_	TiO_2_@PPy–GN	102.2% (50 ppm)	1 ppm	36 s	16 s		[77]
CO	Na-X/rGO/PPy	14.9% (5 ppm)	-	600 s	358 s		[134]
